# Evaluating the ability of AI chatbots to provide informed consent information for common oncological surgeries

**DOI:** 10.1308/rcsann.2025.0089

**Published:** 2025-12-15

**Authors:** RS Sidhu, A Selvamogan, M Abdellatif, R Franscois, A Boddy

**Affiliations:** ^1^ Royal Berkshire NHS Foundation Trust, UK; ^2^ University Hospitals of Leicester NHS Trust, UK

**Keywords:** Artificial intelligence, Readability, Surgical oncology, Informed consent, Information quality

## Abstract

**Introduction:**

Informed consent is fundamental to oncological surgery, but communication is often hindered by medical terminology, inconsistent explanations and variation in patient understanding. Large language models may improve accessibility by generating simplified consent information. This study assessed whether four leading artificial intelligence (AI) chatbots, ChatGPT (GPT-4), Gemini (2.5 Flash), DeepSeek (R1) and Grok (3) could generate information understandable to patients and comprehensive enough to support informed consent for six common oncological operations.

**Methods:**

Standardised patient-style prompts were applied, and chatbot outputs were evaluated for readability using the Flesch Reading Ease Score (FRES), Flesch–Kincaid Grade Level (FKGL) and Gunning Fog Index (GF). Quality and completeness, including coverage of procedure details, risks, benefits, alternatives and consequences of no treatment, were assessed by three consultant surgeons using a modified DISCERN instrument.

**Results:**

Gemini produced the highest quality information (mean DISCERN 72.3 ± 3.0), followed by Grok (63.0 ± 1.8), whereas ChatGPT (48.0 ± 4.7) and DeepSeek (47.1 ± 1.8) performed less well. DeepSeek generated the most readable content (FKGL 9.7; GF 10.8), although no model achieved the recommended sixth-grade level. Common limitations included the lack of systematic referencing (except Gemini), occasional factual inaccuracies, reliance on predominantly US-based resources, and failure to assess patient understanding.

**Conclusion:**

Overall, AI chatbots can provide structured, accessible information to support surgical consent, but current limitations restrict their use as standalone tools. Gemini demonstrated the strongest balance of readability and quality, yet all models require refinement to improve reliability, equity, and patient safety. At present, AI should complement, rather than replace, clinician-led consent discussions.

## Introduction

Informed consent is a cornerstone of ethical medical practice, particularly in the context of oncological surgery, where treatment decisions are often complex, emotionally charged and associated with significant risk.^1^ The informed consent process aims to empower patients by ensuring they understand the nature of the proposed operation, along with its risks, benefits and available alternatives. In oncology, this process is especially critical because of the high-stakes nature of surgical interventions, the urgency of decision making and the potential for life-altering consequences.^2^

Despite its importance, the quality of informed consent remains highly variable. Numerous studies have highlighted deficiencies in how risks and benefits are communicated to patients, citing issues such as inconsistent delivery of information, limited time for discussion and language that is too complex for the average patient to understand.^3^ These problems are compounded in cancer care, where emotions, health literacy and cognitive burden may affect patient comprehension and recall.^4^ Vulnerable populations, including those with lower education levels or non-native language backgrounds, may be particularly disadvantaged in this process, exacerbating disparities in healthcare access and outcomes.^5^

To address these challenges, the use of digital tools such as electronic consent platforms has gained popularity. These platforms allow patients to engage with procedure-specific information in their own time and environment, potentially enhancing understanding and reducing errors.^6,7^ However, even electronic materials have been shown to frequently exceed recommended readability levels (e.g. grades 6 to 8) and may still fall short in ensuring true patient comprehension.^8^

Large language model (LLM)-based artificial intelligence (AI) chatbots, such as ChatGPT, Gemini, Grok and DeepSeek, have emerged as novel tools capable of generating natural language responses to medical questions. Their potential to support patient education and supplement informed consent discussions is gaining attention.^7^ These models provide on-demand, conversational explanations tailored to a user’s query and may offer a scalable way to disseminate complex medical information in a more accessible format.^9^ However, concerns remain about the accuracy, quality and readability of AI-generated content, especially in high-risk fields like oncological surgery, where misinformation could have dire consequences.^2^

This study aims to evaluate whether four widely used AI chatbots can generate patient-friendly, high-quality information sufficient for informed consent in the setting of six common oncological surgical procedures. By assessing the readability and quality of chatbot-generated responses using validated tools, we aim to determine their potential role and limitations as adjuncts in the consent process for cancer surgery.

## Methods

The study was a cross-sectional investigation evaluating the quality and readability of AI chatbot-generated responses to standardised consent-related queries for common oncological surgical procedures. Four AI chatbots – ChatGPT GPT-4 (OpenAI), Google Gemini (2.5 Flash), DeepSeek (DeepThink R1) and Grok 3 (xAI) – were assessed. These chatbots were selected to represent a range of leading LLMs available at the time of the study, with Grok included for its integration with xAI’s ecosystem and potential for real-time adaptability.

Six standardised prompts were developed to reflect typical clinical scenarios requiring surgical consent in oncology. Each prompt followed a consistent structure: “I have [type of cancer] and my doctor has recommended [name of procedure]. Provide me with all the information I need to consent for this procedure.” This format was designed to simulate a patient seeking comprehensive preoperative information, encompassing procedure descriptions, benefits, risks, alternatives and quality-of-life impacts. The specific prompts, surgical procedures and indications are outlined in Table 1. The procedures included lower anterior resection for rectal cancer, abdominoperineal resection for low rectal cancer, right hemicolectomy for ascending colon cancer, subtotal gastrectomy for distal stomach cancer, oesophagectomy for oesophageal cancer and Whipple’s procedure (pancreaticoduodenectomy) for pancreatic head cancer.

**Table 1:** Prompts used for evaluating AI chatbot responses on informed consent for oncological surgeries

Prompt number	Surgical procedure	Indication	Consent prompt
1	Lower anterior resection (LAR)	Rectal cancer	I have rectal cancer and my doctor has recommended a lower anterior resection. Provide me with all the information I need to consent for this procedure
2	Abdominoperineal resection (APR)	Low rectal cancer	I have a low rectal cancer and my doctor has recommended an abdominoperineal resection. Provide me with all the information I need to consent for this procedure
3	Right hemicolectomy	Ascending colon cancer	I have ascending colon cancer and my doctor has recommended a right hemicolectomy. Provide me with all the information I need to consent for this procedure
4	Subtotal gastrectomy	Distal stomach cancer	I have distal stomach cancer and my doctor has recommended a subtotal gastrectomy. Provide me with all the information I need to consent for this procedure
5	Oesophagectomy	Oesophageal cancer	I have oesophageal cancer and my doctor has recommended an oesophagectomy. Provide me with all the information I need to consent for this procedure
6	Whipple’s procedure (pancreaticoduodenectomy)	Pancreatic head cancer	I have pancreatic head cancer and my doctor has recommended a Whipple’s procedure. Provide me with all the information I need to consent for this procedure

The table provides an overview of the six patient-centred prompts administered to each of the four AI chatbots. Each prompt corresponds to a common oncological surgical procedure, including its indication and the exact query phrased as a patient’s request for consent information.

Each chatbot was queried with all six prompts on the same day, 18 July 2025, to eliminate temporal variation in model outputs. Responses were anonymised and compiled into Microsoft Word documents. All chatbot responses were anonymised and randomised before being handed to assessors.

### Readability assessment

The readability of the chatbot responses was assessed using three validated metrics: the Flesch Reading Ease Score (FRES), Flesch–Kincaid Grade Level (FKGL) and Gunning Fog (GF) index.^10,11^ These metrics evaluate text complexity based on sentence length and syllable density, providing insights into comprehension ease for general audiences, which is essential for patient-facing health information. Table 2 outlines the FRES, FKGL and GF scoring criteria. These metrics have been widely used to evaluate the readability of AI-generated responses in medical contexts.^12,13^

**Table 2:** Readability metrics and age-based comprehension levels

FRES score	Gunning Fog index	FKGL	Readability level	US school grade (approx.)	Typical age (years)
90–100	4–6	4–5	Very easy	Grade 4–5	9–10
80–90	6–8	5–6	Easy	Grade 6	11–12
70–80	8–10	6–7	Fairly easy	Grade 7	12–13
60–70	10–12	7–9	Standard/plain English	Grade 8–9	13–15
50–60	12–14	9–11	Fairly difficult	Grade 10–11	15–17
30–50	14–18	11–13	Difficult	Grade 12–14 (high school/college)	17–19+
Below 30	18+	13+	Very difficult	College graduate/professional	21+

This table outlines the Flesch Reading Ease Score (FRES), Gunning Fog index and Flesch–Kincaid Grade Level (FKGL), alongside their corresponding readability levels, descriptions and approximate age for comprehension, according to the US education system.

Responses were processed using the WebFX Readability Test Tool, an online calculator that analyses sentence length and syllable count.^14^ Text was cleaned to remove non-linguistic elements (e.g. bullet points, hyperlinks) before input. Scores were computed for each response.

### Quality assessment

Response quality was assessed using a modified DISCERN instrument, a validated tool for evaluating the for assessing AI healthcare information.^15–17^ The modified DISCERN criteria, detailed in Appendix 1 (available online) include 16 items scored on a 1–5 Likert scale (1 = no/serious shortcomings, 5 = yes/minimal shortcomings), covering clarity of aims, sources, relevance, balance, uncertainty, procedure details, benefits, risks, no-procedure consequences, quality-of-life impact, alternatives and shared decision making (SDM) support. Total scores range from 16 to 80, categorised as excellent (63–80), good (51–62), fair (39–50), poor (27–38) or very poor (16–26).^18^

Three independent assessors, general surgeons with expertise in colorectal and upper gastrointestinal surgery, scored each response whilst blinded to chatbot identities.

### Statistical analysis

All analyses were conducted using R Studio (version 4.5.1, released 13 June 2025) on 29–30 July 2025, with packages including dplyr (version 1.1.4), tidyr (included via namespace), irr (included via namespace), ggplot2 (version 3.5.2) and knitr (version 1.50) for data manipulation, testing, visualisation and table generation. A significance level of *p *< 0.05 was used. The study adhered to ethical guidelines for AI evaluation; no human participants were involved beyond assessors, who provided informed consent.

## Results

The study generated 24 responses (4 chatbots × 6 prompts), which were assessed for quality using the Modified DISCERN instrument and for readability using the FRES, FKGL and GF metrics.

### Inter-rater reliability

Inter-rater reliability for DISCERN scores was excellent (intraclass correlation coefficient = 0.95, 95% confidence interval 0.883–0.979, *F*(23, 21.1) = 26, *p *< 0.001), indicating strong agreement among the three clinicians. Given this high level of agreement, the mean of the three raters’ scores was used for subsequent analyses.

### Quality assessment (DISCERN scores)

The quality results for each model across the six general surgery prompts are summarised in Table 3. Gemini achieved the highest mean (sd) DISCERN score (72.3 ± 3.01), followed by Grok (63.0 ± 1.76). ChatGPT and DeepSeek had lower and similar mean scores of 48.0 ± 4.69 and 47.1 ± 1.84, respectively.

**Table 3:** Mean and standard deviation (sd) of DISCERN scores across AI models

Chatbot	DISCERN (mean)	DISCERN (sd)
Gemini	72.3	3.01
Grok	63.0	1.76
ChatGPT	48.0	4.69
DeepSeek	47.1	1.84
*p*-value	0.0009	–

The table provides a summary of quality metrics for AI-generated responses to prompts on common oncological surgeries. Data represent the average of scores from three independent clinician raters. DISCERN scores assess information quality (higher = better; range: 16–80).

Average DISCERN scores were non-normal (Shapiro–Wilk *W* = 0.885, *p *= 0.010), prompting non-parametric analysis. The Friedman test revealed significant overall differences in median DISCERN scores across the four chatbots (chi-squared 16.4 (df = 3), *p *= 0.0009). However, post-hoc pairwise Wilcoxon signed-rank tests with Bonferroni correction showed no significant pairwise differences (all adjusted *p *≥ 0.213), probably because of limited statistical power from the small number of prompts (*n *= 6) (Appendix 2, available online).

The detailed breakdown of average DISCERN scores for individual criteria across the four chatbots is presented in Table 4.

**Table 4:** Average DISCERN criteria scores across chatbots for oncological surgery consent prompts

Criteria	ChatGPT	Gemini	DeepSeek	Grok
Clear aims	3.7	4.1	3.6	4.9
Aims achieved	4.1	4.7	4.1	4.9
What sources of information	0.0	5.0	0.0	0.0
Source recency	0.0	4.5	0.0	0.0
Additional sources	0.0	3.5	0.0	2.7
Is it relevant	4.0	4.7	4.1	4.6
Balanced and unbiased	4.0	4.7	4.0	4.8
Refer to areas of uncertainty	3.7	4.3	3.5	4.6
Describe how surgical procedure works	3.4	4.6	3.6	4.6
Benefits of procedure	3.9	4.6	3.6	4.8
Risks of procedure	3.7	4.7	3.7	4.9
Describes what if no surgical procedure	2.9	3.9	2.8	4.0
How procedure affects quality of life	3.4	4.8	3.4	4.1
Alternative treatments	3.7	4.7	3.6	4.6
Supports shared decision making	3.9	4.8	3.7	4.7
Overall quality	3.5	4.7	3.5	4.9

The table presents the average scores (across three clinician raters and six prompts) for each of the 16 modified DISCERN criteria, evaluating the quality of informed consent information generated by four AI chatbots (ChatGPT, Gemini, DeepSeek, Grok). Scores range from 1 (poor) to 5 (excellent), with higher values indicating better performance in each criterion.

### Readability scores

FRES, FKGL and GF were tested for each chatbot response; the mean scores by each chatbot are summarised in Table 5. The Shapiro–Wilk test confirmed normality for all metrics and chatbots (all *p*-values >0.05) except the FKGL score for DeepSeek (*p *= 0.042).

**Table 5:** Mean (sd) readability scores by chatbot

Chatbot	FRES mean (sd)	FKGL mean (sd)	GF mean (sd)
DeepSeek	44.4 (3.78)	9.7 (0.651)	10.8 (0.981)
Gemini	43.4 (2.08)	11.1 (0.183)	13.6 (0.264)
Grok	38.8 (5.97)	11.1 (1.23)	13.4 (1.85)
ChatGPT	33.2 (6.14)	13.9 (1.76)	15.6 (1.87)

Summary of readability metrics across the four AI chatbots. A higher Flesch Reading Ease Score (FRES) indicates better readability; lower Flesch–Kincaid Grade Level (FKGL) and Gunning Fog index (GF) indicate easier text. Significant differences (*p *< 0.05): FRES, DeepSeek > ChatGPT (*p* = 0.021), Gemini > ChatGPT (*p* = 0.030); FKGL, DeepSeek < ChatGPT (*p* = 0.00016); GF, DeepSeek < ChatGPT (*p* = 0.0026), DeepSeek < Gemini (*p* = 0.0025).

#### Flesch Reading Ease Score

A repeated measures analysis of variance (ANOVA) revealed a statistically significant difference in FRES scores between chatbots (*F*(3,15) = 5.76, *p *= 0.008). Pairwise post-hoc comparisons (paired *t*-tests with Bonferroni adjustment) did not identify any comparison reaching statistical significance at the adjusted alpha level (all *p*-values >0.05).

#### Gunning Fog Index

The repeated measures ANOVA for GF indicated significant differences across chatbots (*F*(3,15) = 9.54, *p *< 0.001). Pairwise post-hoc analyses demonstrated that DeepSeek scored significantly differently from ChatGPT (*p *= 0.012) and Gemini (*p *= 0.005). No other comparisons reached statistical significance.

#### Flesch–Kincaid Grade Level

A Friedman test showed a significant difference among chatbots for FKGL scores (Friedman chi-squared = 14.95, df = 3, *p*=0.002). Post-hoc Wilcoxon signed-rank tests (with Bonferroni correction) showed no pairwise differences that were statistically significant (all *p*-values >0.05).

## Discussion

Gemini achieved the highest quality score (mean DISCERN: 72.3 ± 3.01; excellent category), largely because of its consistent inclusion of references. These references, all of which were legitimate, were drawn from reputable, up-to-date UK and US sources – including the National Center for Biotechnology Information, Mayo Clinic, Cleveland Clinic, Canadian Cancer Society and Cancer Research UK – which earned additional points under the modified DISCERN criteria. By contrast, ChatGPT, DeepSeek and Grok provided no references, consistent with previous reports identifying the absence of citations as a common shortcoming of AI-generated medical content.^19,20^ Without consistent, verifiable sources, such content risks being perceived as less trustworthy or authoritative, potentially undermining patient decision making or discouraging the use of AI tools altogether.^21^

The absence of citations here does not imply these AIs are incapable of providing them. Mohammad-Rahimi *et al* found ChatGPT-3.5 could produce references when explicitly prompted to “Cite evidence where appropriate”.^22^ This methodological contrast underscores the pivotal role of prompt engineering, which can substantially shape the quality and completeness of AI-generated responses.^23^

Informed consent requires that patients receive accurate, evidence-based information about procedures, including their risks, benefits, alternatives and potential outcomes.^24^ Against this standard, Gemini sets a benchmark for transparency by enabling patients and clinicians to cross-check its outputs against timely, credible sources.

However, during the DISCERN scoring process, factual inaccuracies were picked up in specific AI chatbot responses. For instance, ChatGPT incorrectly implied oesophageal involvement in subtotal gastrectomy for distal gastric cancer, an anatomical error as the procedure focuses on the lower stomach, and Grok misstated “rectal feeding tube” for oesophagectomy postoperative nutrition, which typically uses nasogastric, nasojejunal or jejunostomy methods.^25–27^ These errors exemplify how LLMs powering chatbots can generate confident-sounding but erroneous information because of their probabilistic nature, where outputs are derived from pattern-matching in vast data sets rather than true comprehension or causal reasoning, leading to plausible yet fabricated details known as hallucinations.^2,28^ This variability calls for caution in deploying AI for consent processes: without references, AI tools might amplify health disparities, because vulnerable patients (e.g. those with low health literacy) could accept unverified info more readily, potentially leading to uninformed decisions and exacerbated inequities in healthcare access and outcomes.^29^

A detailed analysis of individual category performance reveals that Grok demonstrated notable strengths in key areas critical to informed consent. Specifically, Grok scored highly (averaging above 4 out of 5) across DISCERN categories, including: Balanced and unbiased, Refer to areas of uncertainty, Describe how surgical procedure works, Benefits of procedure, Risks of procedure, Describe what if no surgical procedure, and Alternative treatments. These high scores indicate that Grok provided comprehensive, balanced and patient-centred information, addressing essential elements required for valid consent under UK guidelines, such as those outlined in the Montgomery v Lanarkshire Health Board [2015] USKC 11 ruling, which emphasises disclosure of material risks, benefits, and alternatives.^30,31^ For instance, Grok’s strong performance in describing risks and alternatives supports patient autonomy by equipping individuals with the knowledge needed to weigh options, a cornerstone of SDM. Similarly, its acknowledgement of uncertainties aligns with ethical requirements to present honest, transparent information, fostering trust in the consent process.^32^

In addition, Grok demonstrated potential by recommending additional support services, such as patient advocacy groups and helplines, representing a constructive step toward facilitating SDM, a core element of effective consent.^24^ The General Medical Council emphasises that SDM involves patients as active partners and advocates for open-ended questioning (e.g. “What concerns do you have?”) to elicit values and preferences.^24^ Such resources may promote active patient engagement, potentially improving adherence and satisfaction through collaborative care planning.^33,34^ Advocacy organisations play a pivotal role in raising awareness, enabling participation in SDM, and helping patients navigate options in alignment with personal priorities.^35^ However, Grok’s suggestions were exclusively US-focused (e.g. American Cancer Society), reducing relevance for UK patients and requiring additional prompting for localisation.^6^ This pattern reflects broader training biases toward US-centric data, reinforcing the need for geographically adapted outputs.^9,36^

Notably, none of the evaluated AIs, except for a single instance with ChatGPT, assessed patient understanding or prompted questions. In one of its six outputs, ChatGPT asked whether users would like “Help understanding what your pathology … means”, indicating some attempt to gauge user comprehension. However, this occurred only once, highlighting the variability and inconsistency of AI chatbot responses. Informed consent requires active confirmation of comprehension (e.g. through teach-back), which most outputs failed to provide.^37^ The AI responses in this study predominantly adopt a monologue-style format, limiting opportunities for clarification and failing to facilitate the real-time dialogue necessary to ensure that decisions are informed by patient priorities. Although Grok’s provision of support group links may support SDM indirectly by connecting patients to external resources, it does not substitute for interactive engagement during the consent process.

Readability is a foundational principle in the provision of informed consent for surgical oncological procedures, where the ethical duty to facilitate patient understanding is codified in both National Health Service (NHS) UK and US guidelines.^38^ These standards advocate for written materials to be comprehensible at or below a grade 6 reading level (age 9–11), acknowledging the substantial portion of adults who read at this level and the proven link between low health literacy and poorer clinical outcomes.^29,38^ In this study, the analysis of 24 AI-generated responses across four chatbots revealed significant variability in readability and quality. DeepSeek exhibited the best readability scores overall, with a mean FKGL of 9.7 and GF of 10.8, although the advantage was only statistically significant for the GF. Gemini also performed well, matching high readability with the highest DISCERN quality score. Encouragingly, this result suggest that greater informational depth does not have to compromise clarity. Nonetheless, none of the chatbot outputs achieved the NHS UK benchmark of FKGL ≤6, indicating that even advanced AI models tend to generate consent information at a higher-than-ideal reading level.^29^ These findings highlight a pressing need for further refinement of AI-generated patient education resources to bridge the persistent gap between technological capability and established health literacy standards, ensuring that all patients are fully supported in making informed choices about their care.^12,15^

### Study limitations

Several limitations temper the generalisability of these findings. First, the study’s small sample size (24 responses from 6 prompts) limited statistical power, as evidenced by the non-significant pairwise DISCERN comparisons despite an overall significant Friedman test. This constraint mirrors challenges in early chatbot research, in which limited prompt diversity may underestimate model performance.^3^ Second, the use of simulated prompts rather than real patient interactions may not fully capture the dynamic nature of consent discussions, a gap noted in studies comparing chatbot and physician responses.^16^ Third, the Modified DISCERN instrument, although validated, may not fully address oncology-specific consent nuances, such as risk communication or regional relevance, which could affect score interpretation.^5,18^ For instance, Grok’s US-centric support recommendations highlight how unprompted outputs may overlook geographic context, potentially requiring additional user input for UK patients.^9,36^ In addition, the study did not assess long-term patient outcomes or comprehension, a critical metric in informed consent efficacy.^4^ Rigorous evaluation across diverse patient demographics, supported by longitudinal outcome studies, is essential to ensure equitable implementation and sustained patient trust.^2^ Specific inaccuracies, such as ChatGPT’s procedural error in subtotal gastrectomy and Grok’s misstatement on feeding tubes in oesophagectomy, underscore the risk of unprompted factual errors, though these could potentially be mitigated with refined prompting.^5,9^ Finally, the rapid evolution of AI models means these results may become outdated, as newer versions (e.g. GPT-4) could outperform those evaluated here.^9^ Given this rapid development, continuous monitoring of AI-generated healthcare information is critical to maintain quality, accuracy and safety, promptly identifying emerging risks.^2^

### Future directions

Future research could explore the integration of prompt engineering strategies to enhance AI performance in consent processes, such as mandating references, comprehension checks and localised resources in queries to mitigate inaccuracies and biases observed here. Furthermore, longitudinal studies assessing patient outcomes, satisfaction and equity when using AI-assisted consent tools would provide empirical evidence on their real-world efficacy. Finally, the investigation of fine-tuned LLMs for surgical oncology could address hallucinations and health literacy barriers, ultimately advancing equitable, evidence-based patient education.

## Conclusions

Although the findings of this study demonstrate that AI chatbots can deliver high-quality, well-structured medical information, their current capabilities fall short of the standards required for obtaining informed consent. Key deficiencies include inconsistent citation practices, occasional factual inaccuracies, limited geographical relevance of recommendations, suboptimal readability and the absence of systematic mechanisms to assess patient comprehension or facilitate real-time dialogue. Among the models evaluated, Gemini achieved the highest DISCERN scores, demonstrated readability comparable with other chatbots and exhibited no obvious factual errors. However, it must be emphasised that AI models evolve rapidly, and comparative performance may change with future updates.^2^ Advances in multimodal AI – capable of processing text, speech and non-verbal cues – may eventually enable interactive, patient-centred consent discussions that meet both ethical and legal standards.^7^ Until such safeguards and functionalities are in place, AI chatbots should be regarded as adjuncts to, rather than replacements for, clinician-led consent processes.

## Competing interests

The author/s declare no competing interests.

## Funding

The author/s received no financial support for the research, authorship and/or publication of this article.

## Ethics approval and consent to participate

This study did not require ethical approval or consent to participate.

## Author contributions

**R Sidhu:** Conceptualisation, Data curation, Formal analysis, Methodology, Project administration, Resources, Software, Visualisation, Writing – original draft, Writing – review & editing. **A Selvamogan:** Conceptualisation, Data curation, Formal analysis, Methodology, Project administration, Resources, Software, Writing – original draft, Writing – review & editing. **M Abdellatif:** Formal analysis, Writing – review & editing. **R Franscois:** Formal analysis, Writing – review & editing. **A Boddy:** Formal analysis, Supervision, Writing – review & editing.

## Artificial Intelligence

The author/s declare that no AI was used to conduct the study or prepare the manuscript.

## Supplementary Information

The online version contains supplementary material available at https://doi.org/10.1308/rcsann.2025.0089.
